# Endothelial dysfunction in systemic lupus erythematosus – a case-control study and an updated meta-analysis and meta-regression

**DOI:** 10.1038/s41598-017-07574-1

**Published:** 2017-08-04

**Authors:** Anselm Mak, Nien Yee Kow, Herbert Schwarz, Lingli Gong, Sen Hee Tay, Lieng Hsi Ling

**Affiliations:** 1 Department of Medicine, Yong Loo Lin School of Medicine, National University of Singapore, Singapore, Singapore; 2 Department of Physiology, Yong Loo Lin School of Medicine, National University of Singapore, Singapore, Singapore; 3 University Medicine Cluster, National University Heart Centre, Singapore, Singapore; 4 Department of Cardiology, National University Heart Centre, Singapore, Singapore

## Abstract

Endothelium-dependent flow-mediated dilation (ED-FMD), a biophysical marker of endothelial dysfunction, is apparently impaired in patients with systemic lupus erythematosus (SLE) but such observation is inconsistent. Here, we assessed and compared the brachial artery ED-FMD (baED-FMD) using ultrasonography between SLE patients without cardiovascular disease and healthy controls (HC) matched for age, gender and body mass index. We then performed a comprehensive meta-analysis of case-control studies which compared baED-FMD between SLE patients and HC by determining the effect size of baED-FMD as standardized mean difference (SMD). Factors associated with the effect size were explored by mixed-model meta-regression. Seventy one SLE patients and 71 HC were studied. SLE patients had lower baED-FMD than HC (3.72 ± 2.8% vs 4.63 ± 3.1%, p = 0.032). Meta-analysis of 25 case-control studies involving 1,313 SLE patients and 1,012 HC with the random effects model revealed lower baED-FMD in SLE patients compared to HC (SMD −1.077, p < 0.001). The presence of diabetes mellitus (p = 0.04747), higher diastolic blood pressure (p = 0.044), renal involvement (p = 0.027) and aspirin use (p = 0.001) were associated with more discrepant baED-FMD between both groups. In conclusion, SLE patients naïve of cardiovascular disease have impaired endothelial function. Diabetes mellitus, renal disease and diastolic hypertension are major contributors of endothelial dysfunction in SLE patients.

## Introduction

Systemic lupus erythematosus (SLE) is a multi-systemic autoimmune condition characterized by tissue inflammation and eventually, organ damage and death^1^. The overall survival of patients with SLE has improved over the past 50 years^2^. However, the accumulation of organ damage has been hindering further improvement of survival in patients with SLE over the most recent 30 years^2^. Apart from renal and neuropsychiatric damage, cardiovascular damage in the form of cardiovascular disease has been demonstrated in several large cohorts to be one of the leading causes of mortality and morbidity in patients with SLE^3, 4^.

When atherosclerosis manifests as clinical events such as myocardial infarction, vascular damage is already advanced and extensive, and often irreversible. Detection of vascular damage to effect primary prevention of serious cardiovascular events is therefore desirable. Although surrogates of atherosclerosis such as coronary artery calcifications and thickened carotid intima are detectable in SLE patients by imaging^5, 6^, these pathological vascular alterations are considered to occur relatively late in the atherosclerotic process^7, 8^. Inoue and Node have proposed the concept of “vascular failure” which comprehensively addresses the progressive atherogenic process, from initial exposure of risk factors causing endothelial dysfunction to smooth muscle dysfunction to full-blown atherosclerotic disease^7^. Endothelial dysfunction is considered to be the initial stage in the pathogenesis of atherosclerosis – it has been shown to predict future cardiovascular events even when coronary angiograms are radiologically normal^9^. In contrast to established atherosclerosis, endothelial dysfunction can be reversed if traditional cardiovascular risk factors are treated^10^. Therefore, identification of endothelial dysfunction affords opportunities for intervention to retard the progress of cardiovascular disease in SLE.

One well-recognized method of assessing endothelial function is by measuring endothelium-dependent flow-mediated dilation (ED-FMD). However, the lack of standardization of the methodology and inclusion of patients with comorbidities may yield inconsistent findings, potentially undermining the clinical application of ED-FMD for cardiovascular risk assessment in patients with SLE. Our objectives in this study were therefore two-fold. Firstly, we evaluated if ED-FMD is indeed impaired in SLE patients naïve of cardiovascular disease and its traditional risk factors by comparing brachial artery ED-FMD (baED-FMD) measured using an ultrasound-based technique^7, 8^ to a group of healthy controls (HC) stringently matched for age, gender and BMI Secondly, we aimed to determine if the putative contribution of SLE to endothelial dysfunction is in fact confounded by demographic-, disease- and treatment-related factors which should be identified in future studies. To this end, with an aim to raise statistical power, we performed a comprehensive meta-analysis of baED-FMD in SLE patients versus matched HC by aggregating the data from our current case-control study and those in the literature employing the same method of baED-FMD evaluation. Meta-regression was performed to identify demographic and clinical factors which potentially impact the effect size.

## Methods

### Subject recruitment and clinical assessment

Adult patients (age ≥21) who fulfilled the American College of Rheumatology (ACR) classification criteria for SLE^11^ were recruited from the Lupus Clinic of the National University Hospital (NUH), Singapore. Patients with positive anti-phospholipid antibodies (anti-cardiolipin, anti-β2 glycoprotein 1 IgG/IgM antibodies) and lupus anticoagulant, or acute illness at the time of recruitment, were ineligible. SLE disease activity and disease-related damage of SLE were assessed by the SLE disease activity index (SLEDAI-2K) and Systemic Lupus International collaborating clinics/ACR damage index (SLICC/ACR DI) at recruitment, respectively^12, 13^. Demographic and clinical information such as duration of disease and drug use were retrieved from clinical interviews and electronic medical records. HC matched for age, gender and body mass index (BMI) were recruited for comparison. HC were mainly nurses of the outpatient clinic at the NUH and their relatives. Exclusions which were applied to both SLE patients and HC were pregnancy, a history hypertension, diabetes mellitus, chronic kidney disease (those with serum creatinine level >120 μmol/L), cardiovascular and cerebrovascular diseases, and statin therapy. Written informed consent was obtained from all participants before recruitment. Our local ethics committee – the NHG Domain Specific Review Board approved the study. All methods in this study were carried out in accordance with the principles of the Declaration of Helsinki.

### Assessment of laboratory parameters

After recruitment and clinical interview, 5–8 ml of peripheral venous blood was obtained by trained phlebotomists. The blood was allowed to clot for 30 minutes at room temperature (RT) and serum was obtained by centrifuging the blood samples at 1300 *g* for 10 minutes at RT. Serum samples were aliquoted into 2 ml Eppendorf tubes and stored at −80 °C for subsequent analyses. One aliquot was sent to the NUH Department of Laboratory Medicine for serum C3 and C4, and anti-dsDNA assays by immunoturbidimetry and enzyme-linked immunosorbent assay (ELISA) (BioRad), respectively. Adipocyte fatty acid binding protein (aFABP) which was reported to be correlated with subclinical atherosclerosis in SLE^14^, was determined by a commercially available ELISA kit (Aviscera Bioscience, Inc., Santa Clara, CA, USA) following the manufacturer’s instruction. The detection range was 1.56–100 ng/ml, with intra-assay and inter-assay precision of 4–6% and 8–10%, respectively. As per standard of care, serum total cholesterol (TC) and high-density lipoprotein cholesterol (HDL-c) levels determined by the NUH Department of Laboratory Medicine were obtained for the SLE subjects.

### Assessment of biophysical markers of cardiovascular disease

Endothelial function was assessed by baED-FMD using the Prosound Alpha-10 ultrasound system (Hitachi-Aloka Medical Ltd., Tokyo, Japan) as previously described^15^. In brief, the brachial artery was imaged using a 10 MHz linear array probe steadied by a stereotactic clamp, and eTRACKING software used to position electronic tracking gates at the media-adventitia interface of opposing arterial walls. Radiofrequency signals from the tracked B-mode images permitted measurement of arterial distension in real time to 0.01 mm accuracy. Reactive hyperaemia was induced by inflating a pneumatic cuff (D.E. Hokanson Inc., Bellevue, WA) placed around the proximal forearm to a pressure of 50 mmHg above systolic blood pressure for 5 minutes, followed by rapid deflation of the cuff. Proprietary FMD software provided a continuous graphical display of minute vasodilation from baseline, cuff occlusion, vasodilation and recovery, and automatically calculated parameters at maximum dilation and %baED-FMD. All subjects abstained from food and exercise, caffeine and alcohol for 12, 24 and 48 hours, respectively, before baED-FMD. Patients who were on angiotensin converting enzyme inhibitors for control of proteinuria were advised to stop the medication 36 hours prior to scanning. In addition, female subjects were studied at least 7 days after cessation of their last menstrual period to minimize the effect of progesterone on endothelial reactivity. Carotid intima-media thickness (cIMT) was evaluated by B-mode ultrasonography of the common carotid artery using the same ultrasound equipment, in accordance with American Society of Echocardiography guidelines^16^. All baED-FMD and cIMT measurements were performed by a single experienced technologist (G.L) blinded to demographic, clinical and laboratory data, in a single scanning session.

### Meta-analysis

#### Search strategy

We performed an extensive search using the relevant keywords “endothelial”, “flow”, “dilation”, “dilatation”, “brachial”, “lupus” and “SLE” in various combinations to identify case-control studies published in English in computerized databases including PubMed (1966 to May 2016), Cochrane Central Register of Control Trials (1^st^ quarter of 2016) and Embase (1980 to May 2016). Scientific abstracts from various rheumatology conferences were not included as detailed methodology is usually unavailable and the findings are often preliminary. We also scanned the articles from the bibliographies of the retrieved review articles. Corresponding authors were contacted by e-mails for essential information unavailable in their published manuscripts.

#### Criteria for selection of studies

Observational case-control studies were included if they met the following criteria: (1) baED-FMD was performed and compared in both SLE patients and HC, (2) subjects had no history of clinical cardiovascular and cerebrovascular diseases, (3) the baED-FMD methodology was similar to that described in the Methods section^15^, and (4) published in the English language or had an English translation. Two investigators (K.N.Y and A.M) independently assessed all publications generated for relevance and conformity to these criteria.

### Statistical analysis

#### Case-control studies

Values are expressed as mean ± standard deviation (SD) unless otherwise stated. The Kolmogorov-Smirnov test was used to check for the normality of the data. The Student’s t-test or Mann-Whitney U test was used where appropriate to examine the differences in continuous variables of interest between SLE patients and HC. Relationships between baED-FMD and various demographic and clinical factors were explored by Pearson or Spearman bivariate correlations where appropriate.

Statistically essential data for subsequent meta-analyses (e.g. SD) which were missing in the published papers were estimated by multiple imputations, a statistically acceptable method to handle missing data in meta-analyses^17^. However, missing demographic and clinical data such as age, gender, BMI, duration of illness and medication use were not imputed as this was deemed inappropriate. All statistical analyses including multiple imputations were performed using IBM SPSS statistics (SPSS version 24, Chicago, IL, USA).

#### Meta-analysis

Effect size was pooled as the standardized mean difference (SMD) and the corresponding 95% confidence interval (CI) of baED-FMD as the primary outcome of meta-analysis. Cochran Q-test was used to assess heterogeneity amongst the participating studies and a value of significance at 10% (p < 0.1) was considered statistically significant for heterogeneity^18^. In addition, I^2^, which describes the percentage of total variation across studies as a result of heterogeneity, was used to detect heterogeneity. Arbitrarily if I^2^ was >40, the random effects model suggested by DerSimonian and Laird was used^19^. For models with statistically significant heterogeneity, meta-regression analyses were performed to identify demographic and clinically-related factors that might contribute to heterogeneity. Mixed-model meta-regression was used because the covariates selected would not be expected to explain heterogeneity of the studies overall^20^. The regression coefficients and the associated standard error (SE), the z score, degree of freedom (*df*), and p values were reported for the meta-regression analysis. Publication bias was assessed by Egger’s regression and reported with Funnel plot with standard error against SMD. All statistical analyses involved in this meta-analysis were carried out with the use of the Comprehensive Meta-analysis Programme, Version 2 (Biostat, Englewood, NJ, USA). To ascertain the quality of the meta-analysis, the MOOSE (meta-analysis of observational studies in epidemiology) and QUOROM (quality of reporting meta-analysis) guidelines were followed where appropriate^21, 22^.

#### Assessment of quality of the case-control studies

The quality of the selected case-control studies was rated with the use of the Newcastle-Ottawa assessment scale designed for assessing the quality of case-control studies for systematic reviews and meta-analyses^23^. Studies were evaluated based on a “star system” in the domains of “selection”, “comparability” and “exposure”^23^. The total score for study quality ranges from the lowest of 0 to the maximum of 9 according to the study quality. While there is no validated cutoff value to discern between studies of good and poor qualities, studies with a score of ≥7 were arbitrarily defined as having a high quality^24^.

#### Sensitivity analyses of meta-analysis

We performed two sensitivity analyses of our meta-analyses. First, we excluded studies with missing data that are essential for synthesizing the effect size. Second, we eliminated studies of low quality from the meta-analysis as assessed by the Newcastle-Ottawa assessment scale as described. The statistical method of effect size synthesis in both sensitivity analyses did not differ from that described in the meta-analysis for the primary outcome.

## Results

### Case-control study

Seventy one SLE patients and 71 matched HC were studied, and there were 6 men in each group. Table 1 summarizes their demographic, clinical, serological and biophysical cardiovascular parameters. The mean ± SD age, BMI and atherogenic index (TC/HDL-c) of SLE patients and HC were 39.21 ± 13.4 and 40.37 ± 12.9 years, 22.54 ± 5.1 and 22.86 ± 4.2 kg/m^2^, and 3.09 ± 1.6 and 3.21 ± 1.4 (p = 0.611), respectively (see Table 1). In SLE patients, the mean ± SD daily prednisolone dose, SLEDAI and SLICC were 13.43 ± 14.4 mg, 6.52 ± 5.4 and 0.17 ± 0.4, respectively. SLE patients had significantly lower baED-FMD than HC (3.72 ± 2.8% vs 4.63 ± 3.1%, p = 0.032) while no difference in cIMT was shown between the two groups (0.56 ± 0.1 vs. 0.56 ± 0.1 mm, p = 0.872). Serum aFABP was significantly higher in patients with SLE than that of HC (14.82 ± 3.3 vs. 13.69 ± 4.6 ng/ml, p = 0.015). In the SLE group, there was no association between baED-FMD and age, gender, BMI, serum C3, C4, blood pressure, atherogenic index, duration of SLE, anti-dsDNA and aFABP levels, SLEDAI, SLICC/DI, atherogenic index, daily prednisolone dose or cIMT (data not shown). Similarly, no association was noted between baED-FMD and age, gender, BMI, cIMT and atherogenic index in the HC group (data not shown).

**Table 1:** Demographics, clinical characteristics, serological and arterial biophysical parameters of patients with SLE and healthy controls.

	SLE patients, (n = 71)	Healthy controls, (n = 71)	P value
	Mean ± SD; number (%)		
Age, years	39.21 ± 13.4	40.37 ± 12.9	—
Gender, female	65 (91.5)	65 (91.5)	—
Body mass index, kg/m^2^	22.54 ± 5.1	22.86 ± 4.2	0.834
Systolic blood pressure, mmHg	122.98 ± 20.6	117.53 ± 15.1	0.076
Diastolic blood pressure, mmHg	73.31 ± 11.9	70.52 ± 10.0	0.133
Atherogenic index*	3.09 ± 1.6	3.21 ± 1.4	0.611
Cumulative clinical manifestation			
Malar rash	38 (53.5)	—	—
Discoid	3 (4.2)	—	—
Photosensitivity	21 (29.6)	—	—
Mucocutaneous ulcers	13 (18.3)	—	—
Arthritis	40 (56.3)	—	—
Serositis	7 (9.9)	—	—
Renal	21 (29.6)	—	—
Neurological	9 (12.7)	—	—
Haematological	32 (45.1)	—	—
Leucopenia	18 (25.4)	—	—
Thrombocytopenia	9 (12.7)	—	—
Anaemia	26 (36.6)	—	—
Haemolytic anaemia	8 (11.3)	—	—
Serology			
ANA positivity	70 (98.6)	—	—
C3, mg/dL	78.73 ± 32.4	—	—
C4, mg/dL	15.16 ± 11.5	—	—
Anti-dsDNA, IU/ml	100.36 ± 88.1	—	—
Duration of disease, months	43.85 ± 62.1	—	—
Prednisolone dose, mg/day	13.43 ± 14.4	—	—
SLEDAI	6.52 ± 5.4	—	—
SLICC/ACR DI	0.17 ± 0.4	—	—
Arterial biophysical parameters			
baED-FMD, %	3.72 ± 2.8	4.63 ± 3.1	0.032
cIMT, mm	0.56 ± 0.1	0.56 ± 0.1	0.872
aFABP, ng/ml	14.82 ± 3.3	13.69 ± 4.6	0.015

Abbreviations: aFABP, adipocyte fatty acid binding protein; SLE, systemic lupus erythematosus; SD, standard deviation; SLEDAI, Systemic Lupus Erythematosus Disease Activity Index; SLICC/ACR DI, Systemic Lupus International collaborating clinics/ACR damage index; baED-FMD, brachial artery endothelium-dependent flow-mediated dilation; PWV, pulse-wave velocity; AI, augmentation index; cIMT, carotid intima media thickness.

*Serum total cholesterol/HDL-c.

### Meta-analysis

**Figure 1:**
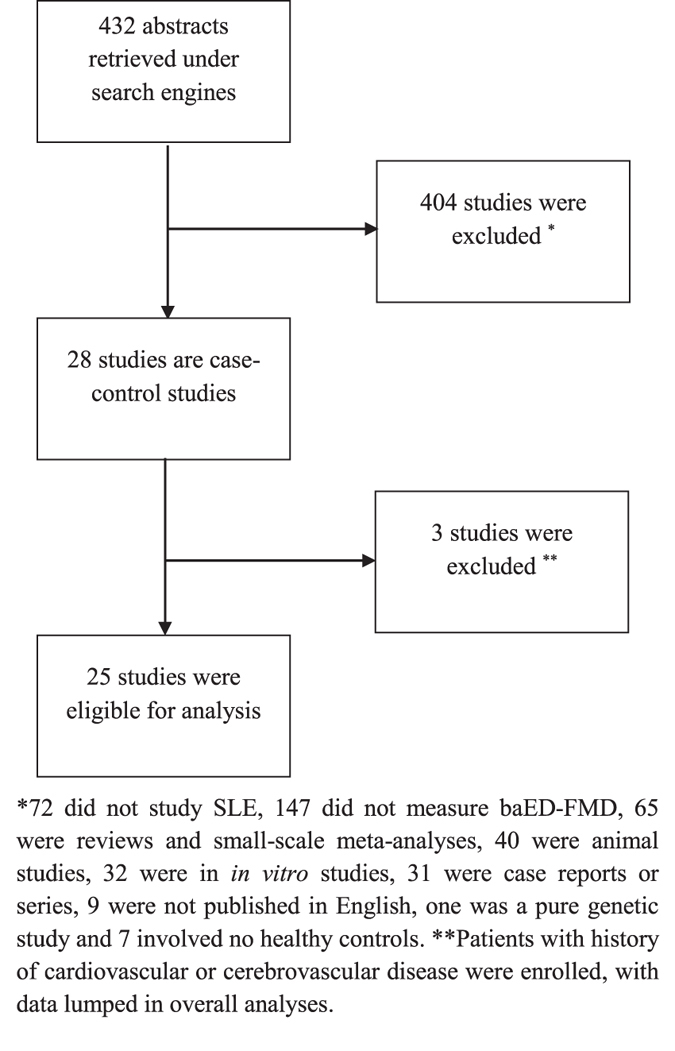
Summary of literature search.

Figure 1 shows the summary of the literature search. 432 abstracts were retrieved under various search engines with 407 of them excluded as the studies assessed non-SLE patients (n = 72), did not evaluate baED-FMD (n = 147), were reviews and small-scale meta-analyses (n = 65), were animal (n = 40) and *in vitro* studies (n = 32) and case reports or series (n = 31), and were not published in English (n = 9). In addition, one pure genetic study, seven studies that did not recruit HC and three which included SLE patients with cardiovascular and cerebrovascular diseases with their data lumped in overall analyses were not selected for meta-analysis.

**Figure 2:**
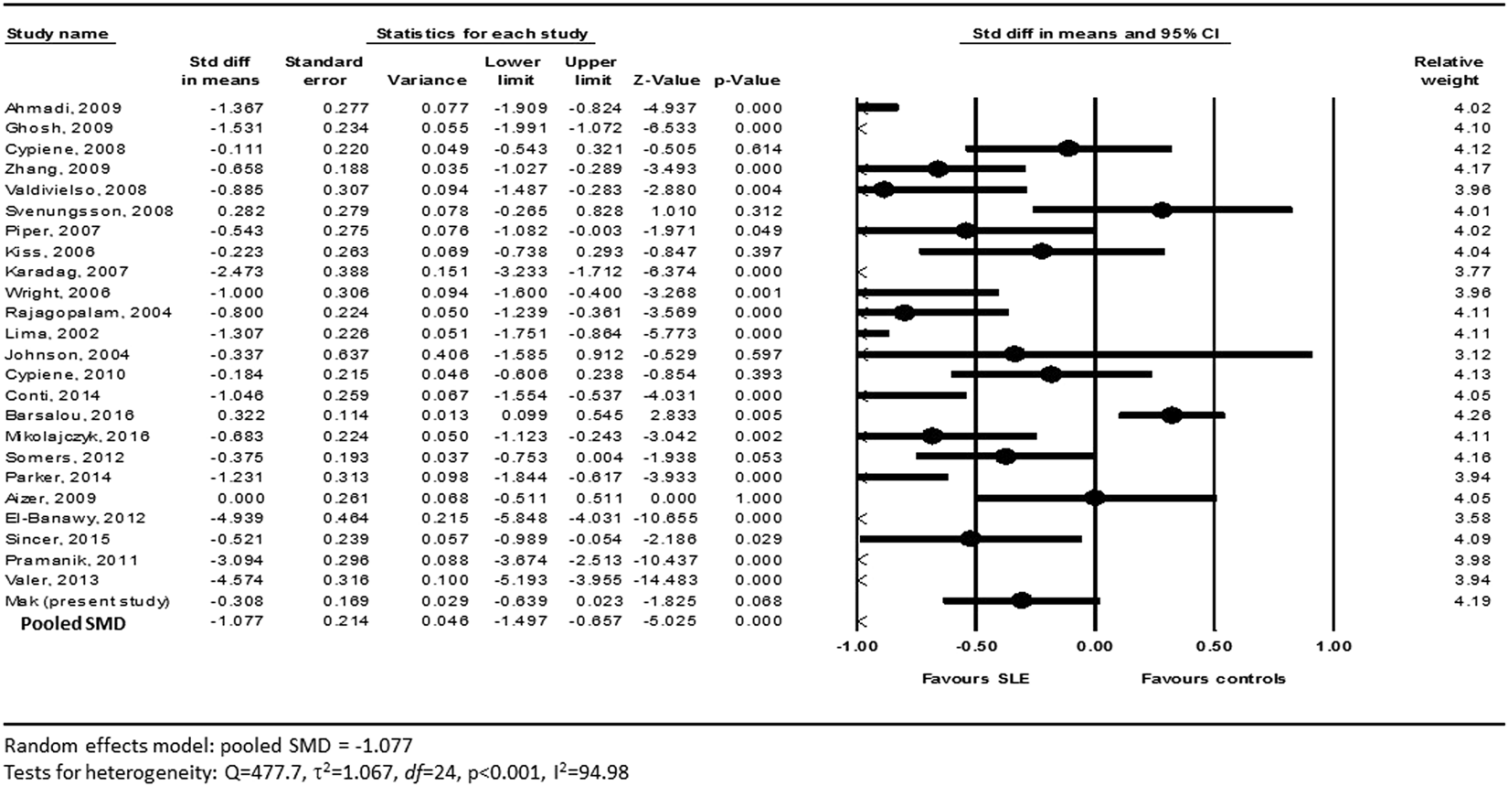
Forest plot of the primary outcome of meta-analysis.

**Figure 3:**
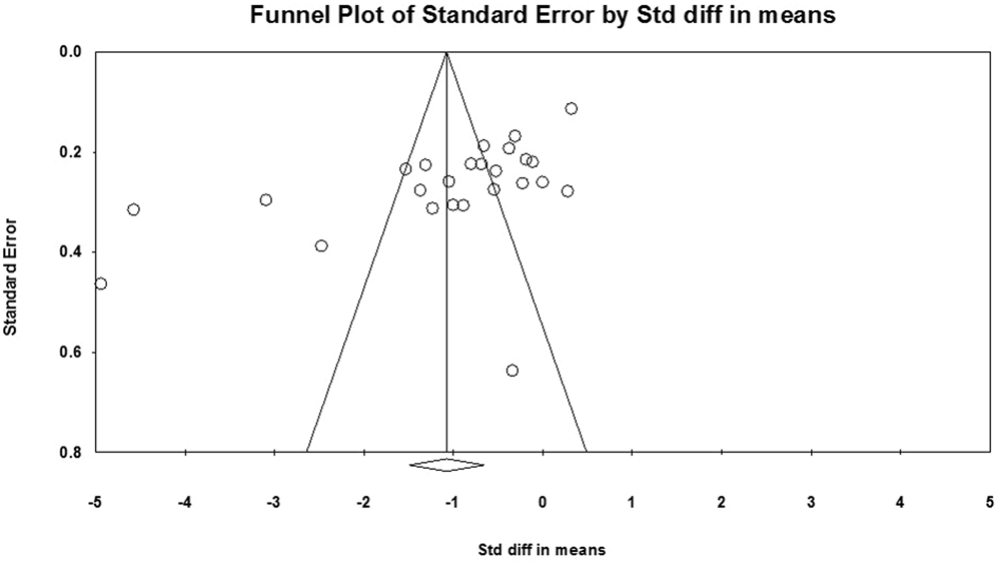
Funnel plot of publication bias of meta-analysis (standard error against standardized mean difference).

After exclusion of ineligible studies and inclusion of data from our case-control study, data from 25^25–48^ studies consisting of 1,313 patients with SLE and 1,012 healthy subjects were pooled for the aggregated effect size of the difference between SLE patients and healthy subjects with respect to baED-FMD (see Table 2). Since a substantial degree of heterogeneity amongst studies was identified by Cochran’s Q and I^2^ statistics (Q = 477.7, I^2^ = 94.98), the random effects model was used for the meta-analysis of the primary outcome. The effect size of aggregated baED-FMD was found to be significantly lower in patients with SLE than in HC (SMD −1.077, Q = 477.7, τ^2^ = 1.067, *df* = 24, 95% CI −1.497 to −0.657, p < 0.001) (see Fig. 2). Publication bias was statistically significant based on Egger’s regression test (intercept = −8.71948, SE = 2.22196, t = 3.9423, *df* = 23, 2-tailed p-value = 0.00068) (see Fig. 3).

**Table 2:** Characteristics of studies comparing brachial artery endothelium-dependent flow-mediated dilation in patients with systemic lupus erythematosus and matched healthy controls.

Author, year	Patients with systemic lupus erythematosus					Matched healthy control				Newcastle-Ottawa Scale			
	Mean age, year	N	Female, %	Disease duration, month	EDD ± SD, %	Mean age, year	N	Female, %	EDD ± SD, %	Selection	Comparability	Exposure	Total
Ahmadi B, 2009	29.6	84	100	68.4	8.50 ± 5.01*	26.5	18	100	15.84 ± 6.88	3	2	2	7
Cypiene A^26^	37.3	30	100	96.12	9.25 ± 5.15	37.45	66	100	9.69 ± 3.29	3	1	2	6
Ghosh P^27^	31	60	90	60	9.97 ± 5.51	34	38	86.8	18.97 ± 6.42	3	2	2	7
Johnson SR^28^	47.1	5	100	198.8	9.62 ± 5.54	42.4	5	100	11.08 ± 2.63	2	1	2	5
Karadag O^29^	40	25	100	90	7.10 ± 2.10	38	22	100	11.40 ± 1.20	3	1	2	6
Kiss E^35^	41.15	33	85.2	122.4	8.81 ± 5.28	48.54	26	84.6	9.86 ± 3.87	1	2	2	5
Lima DS^33^	29	69	100	NR	5.00 ± 5.00	29	35	100	12.00 ± 6.00	3	2	2	7
Piper MK^25^	40.6**	36	100	120	5.60** ± 4.41*	46.0**	22	100	8.00** ± 4.43*	2	2	2	6
Rajagopalan S^36^	37	43	100	NR	3.70 ± 3.50	35	43	50	6.50 ± 3.50	2	2	2	6
Svenungsson E^32^	52.2	26	100	240	6.40 ± 4.20	52.3	26	100	5.10 ± 5.00	4	2	2	8
Valdivielso P^31^	34	26	96.2	NR	12.49 ± 4.47	35	21	95.2	16.91 ± 5.58	3	2	2	7
Wright SA^34^	45	32	88	180	2.40** ± 3.71*	40	19	79	5.80** ± 2.78*	3	2	2	7
Zhang CY^30^	34.4	111	100	112.8	10.87 ± 5.42	34.5	40	100	14.23 ± 4.11	3	1	2	6
Cypiene A, 2010	37.2	31	100	NR	8.95 ± 5.32	37.4	72	100	9.68 ± 3.24	3	1	2	6
Conti F^39^	40	50	88	118.8	6.50 ± 6.6	42.5	25	84	14.4 ± 9.2	3	2	2	7
Barsalou J^40^	17.2	145	83	38.4	8.70** ± 4.19*	14.7	170	55	7.4** ± 3.90*	4	1	2	7
Mikolajczyk TP^41^	44	42	86	NR	9.71** ± 5.54*	41	42	88	13.5** ± 5.55*	3	2	2	7
Somers EC^42^	37.6	95	97.9	NR	4.0 ± 4.7	39.3	38	1	5.7 ± 4.1	3	2	2	7
Parker B, 2013	41.5	27	96	84	1.63** ± 2.41*	38.5	22	86	5.49** ± 3.84*	4	1	2	7
Aizer J^44^	48.8	28	100	194.4	12.50 ± 5.1	47.7	31	100	12.5 ± 4.5	3	1	2	6
El-Banawy HS^45^	27**	60	90	NR	10.0** ± 3.72*	30**	21	85.7	30.5** ± 5.22*	2	1	2	5
Sincer I^46^	37.2	34	56	65	8.10 ± 4.9	35.9	39	53.8	10.6 ± 4.7	3	1	2	6
Pramanik A, 2011	26.1	50	94	75	2.57 ± 2.32	27.8	50	92	8.71 ± 1.58	3	2	2	7
Valer P^48^	41.5	100	92	NR	3.65 ± 1.29	41.4	50	90	10.83 ± 2.02	3	1	2	6
Mak A, 2016	39.2	71	91.5	43.8	3.72 ± 2.8	40.4	71	91.5	4.63 ± 3.1	4	2	2	8

Abbreviations: N, number; EDD, endothelium-dependent dilation at brachial artery; SD, standard deviation; EID, endothelium-independent dilation at brachial artery; NR, not reported.

*Estimated by multiple imputation with 1,000 imputations; **median.

Mixed-model meta-regression revealed that the presence of diabetes mellitus (r = −15.81709, p = 0.04747), higher diastolic blood pressure (DBP) (r = −0.04573, p = 0.04419), renal involvement (r = −4.89258, p = 0.02721) and aspirin use (r = −2.17895, p = 0.00119) in SLE patients were associated with a more discrepant baED-FMD between the SLE and HC (see Table 3).

**Table 3:** Mixed-model meta-regression analysis of potential moderators of difference in brachial artery endothelium-dependent flow-mediated dilation.

	Regression coefficient (SE)	z-score	df	p-value
Age, year	0.004459(0.03261)	1.36791	24	0.172
Female,%	0.04538(2.70484)	0.01678	24	0.987
DM, %	−15.81709(7.98021)	−1.98204	19	0.047
HT, %	−0.46715(1.74889)	−0.26711	16	0.789
Smoking, %	0.59631(1.17388)	0.50798	15	0.611
Menopause, %	1.48237(1.01263)	1.46388	9	0.143
BMI, kg/m^2^	0.16425(0.15941)	1.03039	18	0.303
SBP, mmHg	−0.02230(0.02256)	−0.98834	16	0.323
DBP, mmHg	−0.04573(0.02272)	−2.01229	15	0.044
TC, mg/dL	−0.00495(0.00874)	−0.56697	20	0.571
HDL, mg/dL	0.01665(0.02836)	0.58723	18	0.557
LDL, mg/dL	0.00796(0.01714)	0.46419	16	0.643
TG, mg/dL	−0.00188(0.00909)	−0.20739	16	0.836
ESR, mm/hr	−0.08544(0.04023)	−2.12382	6	0.034
CRP, U/L	−0.00351(0.07112)	−0.04930	12	0.961
SLEDAI, unit	−0.07136(0.05891)	−1.21141	15	0.226
Renal involvement, %	−4.89258(2.21542)	−2.20843	10	0.027
Prednisolone use, %	−0.32784(1.26973)	−0.25820	17	0.796
Mean prednisolone dose, mg/day	−0.07484(0.06059)	−1.23531	12	0.217
HCQ use, %	−1.10975(1.48537)	−0.74712	17	0.455
Aspirin use, %	−2.17895(0.67208)	−3.24208	6	0.001
ACA positivity, %	−0.08995(2.4885)	−0.03688	12	0.971
APASx, %	−2.47656(3.01813)	−0.82056	4	0.412
Disease durations, months	0.00510(0.0382)	1.33454	16	0.182

Abbreviations: SE, standard error; *df*, degree of freedom; DM, diabetes mellitus; HT, hypertension; BMI, body mass index; SBP, systolic blood pressure; DBP, diastolic blood pressure;TC, total cholesterol; HDL, high density lipoprotein; LDL, low density lipoprotein; TG, total triglyceride; ESR, erythrocyte sedimentation rate; CRP, C-reactive protein; SLEDAI, Systemic Lupus Erythematosus Disease Activity Index; HCQ, hydroxychloroquine; ACA, anti-cardiolipin antibodies; APASx, antiphospholipid antibody syndrome.

The missing SD of baED-FMD in seven out of 25 studies necessitated imputation (see Table 2). After removing these 7 studies with missing SD of baED-FMD from the meta-analysis, there was no change in the significance and direction of the effect size of the primary outcome (SMD −0.999, 95% CI −1.478 to −0.521, p < 0.001). With respect to study quality, after removal of 12 low-quality studies based on the Newcastle-Ottawa assessment scale, the effect size of aggregated baED-FMD remained significantly lower in patients with SLE than in HC (SMD −0.926, 95% CI −1.414 to −0.438, p < 0.001) (see Table 4)

**Table 4:** Results of sensitivity analyses based on quality of studies and exclusion of studies with missing data.

	Number of studies involved	Pooled SMD (95% CI)
Quality of studies		
All studies	25	−1.077 (95% CI −1.497, −0.657)^†^
*High-quality studies	13	−0.926 (95% CI −1.414, −0.438)^†^
Missing data		
All studies	25	−1.077 (95% CI −1.497, −0.657)^†^
**Studies without missing data	18	−0.999 (95% CI −1.478, −0.521)^†^

Abbreviations: SMD, standardized mean difference; CI, confidence interval.

* Newcastle-Ottawa quality assessment total score ≥7. **Exclusion of Ahmadi, *et al*.^37^, Piper, *et al*.^25^, Wright, *et al*.^34^, Barsalou, *et al*.^40^, Mikolajczyk, *et al*.^41^, Parker, *et al*.^43^ and El-Banawy, *et al*.^45^. ^†^p < 0.001.

## Discussion

Owing to the advent of non-invasive ultrasonic imaging techniques, endothelial dysfunction has been increasingly recognized in patients with SLE over the past 2 decades or so^49^. Lupus-related factors such as inflammation, immune dysregulation, renal involvement and glucocorticoid use putatively contribute to impaired endothelial function but traditional cardiovascular risk factors are also prevalent in patients with SLE. A key question is whether endothelial dysfunction is caused primarily by SLE *per se*, its therapy or associated comorbidities. If the former is operative, longitudinal screening of endothelial function in SLE patients naïve of cardiovascular disease may be warranted, especially those in whom disease-related factors detrimental to endothelial health can be identified.

In this case-control study of SLE patients free of clinical cardiovascular disease and stringently-matched HC, both with identical and on average, normal cIMT, we observed worse endothelial function as assessed by baED-FMD in SLE. Because of the modest sample size with potential lack of statistical power in the current case-control studies and those in the literature, we attempted to increase the statistical power by aggregating our data with those available in the literature using meta-analysis, and by performing meta-regression to identify associations between endothelial dysfunction and demographic, serological as well as disease-related factors. Data from over 1,300 SLE patients and 1,000 matched HC from 25 studies confirmed that SLE patients had inferior endothelial function as compared to matched HC, despite the absence of known cardiovascular disease. In meta-regression analysis, the presence of DM, higher DBP, renal involvement and aspirin use significantly widened the difference in baED-FMD between SLE patients and HC, signifying an association of these factors with poorer endothelial function in SLE.

Apart from the presence of renal lupus, we cannot fully explain in our meta-analysis as to why the association between SLE-related features and the discrepancy in baED-FMD between SLE patients and healthy controls is absent, even though SLE patients had poorer baED-FMD compared to their healthy counterparts was found in our case-control study. There are three possible explanations. First, individual SLE-related feature *per se* may not be sufficiently strong to lead to detectable difference in baED-FMD between SLE patients and HC. Secondly, a combination of various SLE-related and SLE-non-related factors might be required to worsen baED-FMD. Third, we might have simply missed some as-yet unknown factors which contribute to endothelial function in patients with SLE.

In contrast to our study which addresses the issue of endothelial function in patients with SLE, studies of other rheumatic conditions such as rheumatoid arthritis (RA) found that serum C-reactive protein (CRP) level, a marker of RA disease activity, was correlated with endothelial dysfunction in patients with RA^50^. This is not surprising because the pathophysiology of RA is indeed very different from that of SLE. Both ESR and CRP are reasonably good biomarkers of RA disease activity and CRP itself is a predictive marker of cardiovascular disease^51^. In contrast, CRP is not a reliable disease activity marker of SLE. In fact, most patients with SLE do not mount sufficient CRP response when their disease is active. This partly explains why we failed to detect a significant relationship between markers of SLE disease activity and endothelial dysfunction.

The findings of our meta-regression analysis have important clinical implications. First, lupus patients with DM, diastolic hypertension and renal lupus who may be at greater risk of developing endothelial dysfunction, a precursor of frank atherosclerosis, may require closer monitoring and aggressive management for cardiovascular risk factors. Secondly, in the era of preventive medicine, non-invasive screening for endothelial dysfunction coupled with therapeutic lifestyle modification would be an attractive strategy for patients with SLE to reduce future cardiovascular events. In order to address the impact of SLE *per se* on endothelial function, investigators should consider excluding SLE patients with DM, hypertension and lupus nephritis in future prospective studies.

Surprisingly, aspirin use was found to be associated with poorer endothelial function in patients with SLE in our meta-regression analysis. Although counter-intuitive, this may indicate confounding by indication as aspirin may be prescribed for those patients perceived to have higher vascular risk, or are indeed at greater risk on account of antiphospholipid antibodies^52^.

There are several limitations of this study. In the context of SLE, baED-FMD remains a surrogate cardiovascular biomarker of uncertain prognostic significance^53^. Its validity as a useful non-invasive screening tool should be addressed by longitudinal outcome studies in large cohorts. Second, missing data were present in the meta-analyses of the primary outcomes which required multiple imputations, and not all studies were included in the meta-regression analyses. Even though sensitivity analyses did not alter the significance and direction of the primary outcome, the results should be interpreted with caution. In our meta-analysis, the impact of statin use on endothelial function was not assessed. This is because including our present case-control study, most of the studies which aimed to compare endothelial function between SLE patients and HC excluded subjects who used statin. As such, data on statin use is insufficient for meaningful evaluation of the impact of statin therapy on endothelial function by meta-regression analysis. Finally, publication and aggregation biases are invariably present in meta-analyses because they are not based on subjects’ individual data. Indeed, publication bias was statistically significant based on the Egger’s regression test of our meta-analysis. The results of this meta-analysis must therefore be interpreted with caution. Nevertheless, the relative weight was evenly distributed between individual studies which led to the primary outcome, with the weight ranging from 3.12 to 4.26, suggesting that there was no bias in particular studies contributing towards the effect size.

## Conclusion

Patients with SLE who are naïve of cardiovascular disease have impaired endothelial function as determined by baED-FMD. While meta-analysis confirmed that baED-FMD is impaired in patients with SLE without clinically overt cardiovascular disease, the presence of DM, higher diastolic BP and renal involvement potentially contributes to endothelial dysfunction. As such, lupus patients with comorbidities such as DM, diastolic hypertension and renal involvement should deserve more judicious and aggressive monitoring for unfavorable cardiovascular outcomes.
